# A Closer Look to the Evolution of Neurons in Humans and Apes Using Stem-Cell-Derived Model Systems

**DOI:** 10.3389/fcell.2021.661113

**Published:** 2021-04-21

**Authors:** Maria Schörnig, Elena Taverna

**Affiliations:** Max Planck Institute for Evolutionary Anthropology, Leipzig, Germany

**Keywords:** stem cell, model system, primate, neuroscience, brain evolution, comparative neurobiology, iPS cells

## Abstract

The cellular, molecular and functional comparison of neurons from closely related species is crucial in evolutionary neurobiology. The access to living tissue and post-mortem brains of humans and non-human primates is limited and the state of the tissue might not allow recapitulating important species-specific differences. A valid alternative is offered by neurons derived from induced pluripotent stem cells (iPSCs) obtained from humans and non-human apes and primates. We will review herein the contribution of iPSCs-derived neuronal models to the field of evolutionary neurobiology, focusing on species-specific aspects of neuron’s cell biology and timing of maturation. In addition, we will discuss the use of iPSCs for the study of ancient human traits.

## Discovery and Use of iPSCs

The closest living relatives of modern humans are the great apes and among them, chimpanzees and bonobos who’s lineages split from the last common ancestor with humans about 5-10 million years ago (Pääbo, 2014). Historically, comparative studies of human and non-human primate brains have been difficult due to ethical concerns regarding the use of primary tissue, its limited availability and the lack of convincing model systems. To understand the differences between human and non-human primate brains and their cognitive abilities, it is important to study morphological and functional differences at the cellular level. As non-human great apes are acutely endangered and the availability of primary tissue is limited, little can be learned and known from the cell biological comparison to great apes. Encouragingly, the development of *in vitro* systems, including the use of induced pluripotent stem cells (iPSCs), have expanded the range of comparative studies possible. In 2006, Shinya Yamanaka’s research group made a groundbreaking finding: they discovered that murine fibroblasts could be genetically engineered to a pluripotent, stem cell-like state (Takahashi and Yamanaka, 2006). One year later they were successful in reprogramming human fibroblasts (Takahashi et al., 2007).

Similar to embryonic stem cells (ESCs), iPSCs can differentiate into many different cell types, for example neurons (Reubinoff et al., 2001; Zhang et al., 2001), insulin-producing beta cells (Assady et al., 2001), cardiovascular cells (He et al., 2003), hematopoietic cells (Kaufman et al., 2001; Chadwick et al., 2003), and many other cells of the human body (Williams et al., 2012), or self-organize into complex three-dimensional structures containing multiple cell types that resemble human tissues, called organoids (Lancaster and Knoblich, 2014; Clevers, 2016). These stem cell-derived systems can be used to study disease-associated pathomechanisms *in vitro*, test drugs, develop tissue replacement and patient specific therapies and to explore how natural variation between humans and non-human primates impact development, cell biology and disease (Park et al., 2008; Marchetto et al., 2010; Lancaster et al., 2013; Shcheglovitov et al., 2013; Arbab et al., 2014; Hinson et al., 2015; Mora-Bermudez et al., 2016; Frega et al., 2019; Klaus et al., 2019).

In comparison to the use of ESCs and primary tissue, the use of iPSCs poses less ethical concerns because easily accessible somatic cells, like blood cells, keratinocytes, and buccal cells can be harvested and reprogrammed into iPSCs from humans and non-human primates without harming them. iPSCs are the only tool of choice for comparative studies between humans and apes on living tissue, because of the lack of ape ESCs lines and restricted access to primary tissue. Early analysis of iPSCs differentiation could demonstrate that both ESCs and iPSCs follow the same steps and time course during differentiation. However, iPSCs demonstrated a lower efficiency and greater variability when differentiating into neural cells and suggests that individual iPSC lines may be “epigenetically unique” (Hu et al., 2010).

In this review we give an overview of some recent studies in which iPSCs were used to model brain maturation and evolution in humans and non-human primates. Through these examples we will illustrate how iPSCs have been successfully used to: (1) model brain evolution, (2) study human-ape differences in neuronal structure and function and (3) gain insight into brain development of human extinct relatives, such as Neanderthals and Denisovans using CRISPR/Cas genetic engineering.

## Brain Evolution in a Dish

### Cerebral Organoids as a Model System for Human Brain Evolution

By taking up over three-quarters of the human brain, the cerebral cortex is the integrative and executive center of the mammalian central nervous system (Rakic, 2009; Fernández et al., 2016). Differences in cognitive abilities between humans and non-human primates are thought to depend on more complex neural architectures that result from an increased number of neurons and cerebral cortex size in humans (Herculano-Houzel, 2012; Geschwind and Rakic, 2013). Several studies have addressed the cellular and molecular basis of cognitive differences focusing on neurogenesis, the process through which neurons are generated during embryonic development. These studies revealed that humans have expanded proliferative zones and diverse subtypes of neural stem and progenitor cells with enhanced proliferative capacities. All these features have the potential to increase the final number of neurons and facilitate neocortex expansion (Smart et al., 2002; Lui et al., 2011; Borrell and Reillo, 2012; Namba and Huttner, 2017; Kalebic and Huttner, 2020). Interestingly, especially area 10 of the frontal lobe seems to be larger in the human brain compared to the relative frontal lobe size in other apes. Thereby the supragranular layers of area 10 in human brains offer more space available for neuronal connections with other higher-order association areas, suggesting specialized cognitive functions associated with this part of the cortex during hominid evolution (Semendeferi et al., 2001).

The development and differentiation of human and ape iPSCs into diverse neuronal systems made comparative studies between species possible and helped partially fill this gap of knowledge. 2D (e.g., induced neurons) and 3D systems (e.g., brain organoids) of humans and non-human apes have recently emerged as an exciting new experimental model, allowing sophisticated analyses of the early steps of brain development in health and disease and of how development has changed during evolution (Lancaster et al., 2013; Lancaster and Knoblich, 2014; Camp et al., 2015; Otani et al., 2016; Klaus et al., 2019; Pollen et al., 2019; Kyrousi and Cappello, 2020).

**FIGURE 1:**
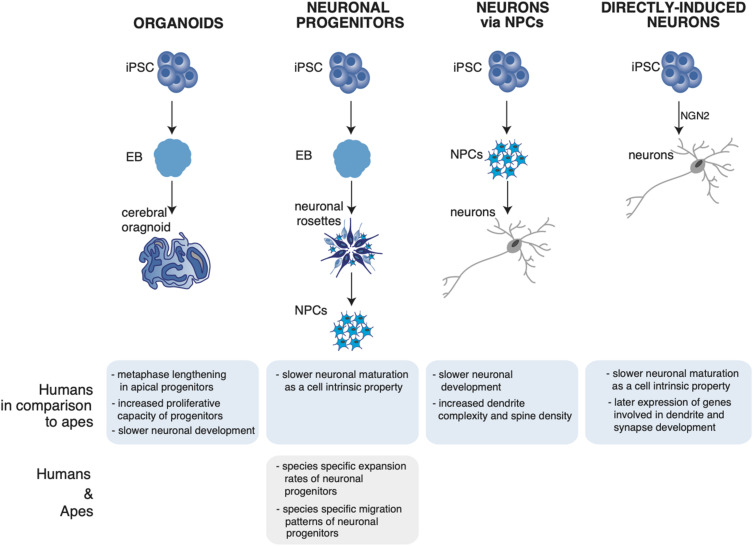
Summary of neuronal phenotypes observed in human and ape neuronal models. The figure shows the differentiation of induced pluripotent stem cells (iPSCs) into cerebral organoids (Mora-Bermudez et al., 2016; Kanton et al., 2019; Pollen et al., 2019; Benito-Kwiecinski et al., 2021), neural progenitors (NPCs) (Otani et al., 2016), neurons via NPCs (Marchetto et al., 2019), and directly induced neurons (Schörnig et al., 2021). **(Bottom)** Observed neuronal phenotypes in the respective neuronal system and their uniqueness in either humans or non-human apes. iPSC, induced pluripotent stem cells; EB, embryoid body; NPC, neuronal progenitor; NGN2, neurogenin-2.

3D systems such as human cerebral organoids were shown to recapitulate fetal brain tissue at the cellular level as they use similar gene networks controlling neural progenitor proliferation and differentiation and similar genetic programs to generate a structured cerebral cortex (Camp et al., 2015). When human cerebral organoids have been compared to cerebral organoids of chimpanzee, they were found to be remarkably similar in terms of their cytoarchitecture, cell type composition, and neurogenic gene expression (Mora-Bermudez et al., 2016). Despite the similarities, human apical progenitors (APs; the founders stem and progenitor cells of the brain) were reported to have a longer prometaphase-metaphase length and a higher proliferative capacity (Mora-Bermudez et al., 2016), suggesting a possible contribution to the increase in the human neocortex size. In line with these findings, a recent report suggests a higher proliferative capacity of human forebrain organoid APs compared to those of gorilla organoids (Benito-Kwiecinski et al., 2021). The human organoids were found to be bigger in size and showed expanded lumens in comparison to the gorilla organoids. The authors explained this observation by a delay, in the human organoids relative to gorilla, in the switch from symmetrically expanding neuroepithelial (NE) cells to neurogenic apical radial glia (aRG) cells. The higher proliferative capacity was directly linked to the ability of human APs to remain longer in a NE-like state (Benito-Kwiecinski et al., 2021) (Figure 1, organoids).

The systematic comparative analysis of human, ape and primate organoids via single cell RNA sequencing (scRNAseq) led to the identification of human-specific features of cortical development. Pollen et al. (2019) identified differentially expressed genes in human organoids enriched for recent gene duplications, including multiple regulators of PI3K-AKT-mTOR signaling possibly contributing to human brain development and evolution. Of note, the mTOR pathway promotes stemness and stem cells self-renewal, and it is therefore an appealing candidate for cortical expansion (Rafalski and Brunet, 2011; Zou et al., 2012). From a cell biological point of view, one feature appeared to distinguish human cerebral organoids from chimpanzee organoids: the length of APs metaphase, that is longer in human proliferating APs compared to chimpanzee (Mora-Bermudez et al., 2016) (Figure 1, organoids).

By analyzing cerebral organoids of human, chimpanzee and macaque using scRNAseq and accessible chromatin profiling Kanton et al. found a slower neuronal development in human organoids relative to primates. In addition, human and chimpanzee cells followed distinct cell states along progenitor-to-neuron lineages, identified by a progression through stem cell states, progenitor cells of multiple brain regions including the forebrain, midbrain, hindbrain, and retina into differentiation of excitatory and inhibitory neurons and astrocytes. The developmental timing of deep and upper layer neurons was found to be different between the species. To understand which human-specific gene-expression patterns observed in the developing cortex persist into adulthood, the organoid expression data were compared to single-nuclei RNA-seq data from post-mortem prefrontal cortex tissues of human, chimpanzee, bonobo and macaque. Some of the differences observed during development persisted into adulthood, as in the case of astrocytes having the largest number of human-specific differentially expressed genes. Other cell-state-specific changes were found that occur exclusively during development (Kanton et al., 2019) (Figure 1, organoids).

These subtle differences in organoid progenitors and neurons between humans and chimpanzees may have consequences for expansion of the human neocortex and suggest a slower development and maturation of human cortical neurons compared to chimpanzee and other primates.

### Studying Human-Ape Differences Using iPSCs Derived Neurons

While cerebral organoids allow researchers to take a closer look to brain development and the generation of neurons, the differentiation from iPSCs toward specific neuronal and glia subtypes [including excitatory and inhibitory neurons, astrocytes, oligodendrocytes, and microglia, see Mertens et al. (2016)] allows to model and study evolutionary aspects of neuronal maturation and neuronal output.

Otani et al. made use of differentiating human, chimpanzee and macaque stem cells in 2D (adherent cells) and 3D (organoid) neuronal systems and show that the expansion of the neuronal progenitors differed between the three species, leading to a different final number of neurons. The control of the cortical neuron number generated by a single progenitor is regulated cell autonomously in culture, suggesting that primate cerebral cortex size might be regulated at least in part at the level of individual cortical progenitors (Otani et al., 2016) (Figure 1, neural progenitors).

The use of iPSCs-derived neurons has offered the unique opportunity to give a closer look at the dynamics of neuronal development and maturation. By using iPSCs-derived human pyramidal neurons researchers have recently found that human pyramidal neurons have an overall slower structural and functional maturation over time, resulting in a higher dendrite complexity and dendritic spine density compared to their chimpanzee counterpart (Marchetto et al., 2019) (Figure 1, neurons via NPCs).

A further refinement in iPSCs-derived neurons allows to obtain neurons directly from iPSCs, without the transition through neuronal progenitor cells (NPCs) (Zhang et al., 2013; Frega et al., 2017; Nehme et al., 2018; Nickolls et al., 2020). In contrast to the differentiation of neurons based on NPCs, direct conversion protocols can be a fast tool to generate neurons by bypassing proliferating NPCs, thus eliminating the influence of cell cycle as a possible confounding factor. When this system was used to study and compare directly maturation and differentiation of human and ape neurons, human neurons were found to develop slower than ape neurons from a functional and transcriptional point of view. Since in this system cell cycle is no longer a confounding factor, the authors suggested that the slower maturation of human neurons is a cell intrinsic property of the human neurons (Schörnig et al., 2021) (Figure 1, directly induced neurons); a summary of the observed phenotypes in human and ape neuronal models is shown in Figure 1.

The findings obtained using iPSCs-derived neurons are compatible with postmortem studies of human and primate brains showing that the human brain develops more slowly than the brain of other primates (neoteny) and suggest that the human brain shows signs of changes in timing and rate of developmental events (heterochrony). Heterochrony and neoteny of the human brain development, in comparison to mouse and non-human primates, has been reported using different experimental systems (primary tissue, organoids, 2D culture, and transplantation experiments) at different levels, ranging from the overall neuronal structure, to dendritic spines and the transcriptomic level (Somel et al., 2009; Petanjek et al., 2011; Zhu et al., 2018 ; Kanton et al., 2019; Linaro et al., 2019).

The heterochrony is considered to have important consequences on the neuron maturation and on neuron’s complexification, as demonstrated for pyramidal neurons (Benavides-Piccione et al., 2006; Defelipe, 2011; Espuny-Camacho et al., 2013; DeFelipe, 2015; Linaro et al., 2019). Comparative Golgi staining showed that pyramidal neurons undergo a prolonged period maturation resulting in a more complex and intricated dendritic tree in humans compared to chimpanzees and other primates (Bianchi et al., 2013a,b). Given the role of pyramidal neurons in the evolution of higher cognitive functions, a higher degree of branching in humans could provide the basis for higher integration and computation’s ability.

### Induced Pluripotent Stem Cells Derived Neurons and the Dawn of Experimental Paleo-Neurobiology

Comparative analyses of human and ape neuronal systems shed light on neuronal changes occurring during evolution and increased the interest in studying evolution on the human lineage in different time windows and in different cell types. The iPSCs-derived brain organoids and neurons offer unique tools to model and study also ancient human neurons and brain (at least in the form of mini-brains/organoids).

The availability of high-quality genomes from ancient hominins (Meyer et al., 2012; Prüfer et al., 2014, 2017; Mafessoni et al., 2020) made it possible to compare the genomes of modern humans and ancient hominids and rose questions about phenotypic changes during evolution between different human species. The comparison of the genomes of present-day people to the genomes of Neanderthals (Prüfer et al., 2014, 2017; Mafessoni et al., 2020) and Denisovans (Meyer et al., 2012) led to the generation of a catalog (Kuhlwilm and Boeckx, 2019; Heide, 2020) of 31,380 single nucleotide substitutions and 4,113 small insertions and deletions that are shared among >99.8% of modern humans and differ from the last common ancestor with Neanderthals and Denisovans (Pääbo, 2014). Genetic engineering tools, like gene editing, make it now possible to study such changes and their involvement in disease and evolution. By editing the genomes of human iPSCs one would be able to systematically study the functional consequences of the genomic changes that set modern humans apart from their closest evolutionary relatives, such as apes and archaic humans [see the recent example of NOVA1 (Trujillo et al., 2021)].

**FIGURE 2:**
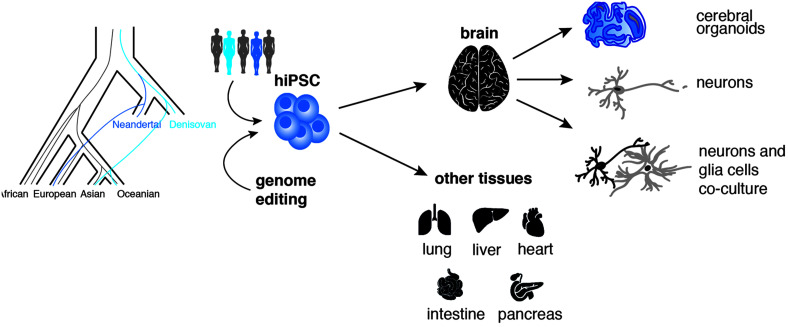
Addressing the neurobiology of recent human history using iPSCs. Human pluripotent stem cells (iPSCs) carrying natural or introduced (via CRISPR-Cas9 genome editing) Neandertal or Denisovan ancestry can be differentiated into various 2D-cell systems and 3D-organoids types to generate “ancestralized” cells and tissues like brain, lung, liver, heart, pancreas, and intestine (Dannemann et al., 2020).

One drawback of genome editing is the limit of introducing many precise nucleotide changes on many different chromosomes at once. So far precise editing is possible for up to four genes simultaneously (on eight chromosomes) in the same cell (Riesenberg et al., 2019). Considering the high number of changes on the human lineage and the possible drawbacks of off-target effects introduced during each step of editing, additional model systems could present themselves as attractive alternatives. One appealing alternative is represented by the use of human iPSCs repositories. Genomics studies showed that a portion of the genome from present-day humans outside Africa originates from an ancient admixture between modern humans and archaic humans. This admixture introduced Neanderthal and Denisovan alleles that are still present in humans today (Meyer et al., 2012; Prüfer et al., 2014; Slon et al., 2018) and that are carried on in human iPSCs derived from present day individuals (Dannemann et al., 2020) (Figure 2).

Induced pluripotent stem cells repositories offer a great potential to explore experimentally human and ancestral functional variation and its contribution to human phenotypes and disease. Recent studies begun to investigate the effects of these introgressed archaic genetic variants on modern human phenotypes such as pain sensitivity (Zeberg et al., 2020a), progesterone receptor expression levels (Zeberg et al., 2020b) and COVID-19 severity (Zeberg and Pääbo, 2020).

The protocols to differentiate iPSCs in neuronal systems make it now possible to study genetic changes exerting effects in the nervous system. For example, one genetic change between modern and archaic humans potentially involved in speech development was identified in the binding site for the transcription factor POU3F2 (Maricic et al., 2013) and could be investigated in differentiated neuronal systems.

## Conclusion and Further Perspectives

To summarize, iPSCs offer a great tool for research on topics where the availability of tissue is a limiting factor, for ethical (apes) or practical reasons (ancient humans). While planning experiments, one should keep in mind the possible limitations of the iPSCs-derived systems. For 3D systems, the proportion of different stem cells types in organoids can differ from primary tissue, possibly for reasons related to the culture conditions (Heide et al., 2018). In addition, the lack of vascularization and the presence of a cell stress status (Pollen et al., 2019) are parameters that might generate differences to the primary tissue. Of note, these parameters have the potential to affect the species comparison. In a recent study, researchers tried to overcome some of these variabilities by fusing human and chimpanzee induced pluripotent stem cells to generate tetraploid hybrid stem cells and in turn hybrid organoids (Agoglia et al., 2021).

For iPSCs-derived neuron systems, one cannot rule out that a fast maturation protocol (as in the case of iNs) could skip crucial step(s) required for precise neuronal fate specification. We and others recently reported a high degree of cell heterogeneity in iNs cultures (Lin et al., 2020; Schörnig et al., 2021). The systematic use of scRNAseq and immunofluorescence for fate markers revealed that in a single iNs culture several fates/identities are represented: central, peripheral, sensory, and pyramidal. Although cell heterogeneity can be beneficial in comparative studies, it might pose challenges in translational research, as the perfect control of cell composition is key for clinical applications. Despite these limitations, iPSCs-derived neuron and organoid protocols allowed to dissect with an unprecedented precision basic principle underlying neuronal development and maturation in phylogeny. *De facto*, the iPSCs revolution allows us to address questions we could not have addressed otherwise. The possibility to control iPSCs to generate different neuron subtypes, and the fact that they are so easily accessible, make them an ideal experimental system for future studies of the evolutionary cell biology of neurons. Since the central nervous system is made not only by neurons, but also by astrocytes and other glial cells that tightly interact with neurons, one should expect that these 2-D systems will be of a great help to model evolutionary differences in astrocytes and/or oligodendrocytes as well. In addition, mixed cultures of iPSCs-derived neurons and glial cells might offer a unique opportunity to study the evolution of cell-to-cell interaction and cross talk in human and ape brains.

## Author Contributions

MS and ET wrote the manuscript. Both authors contributed to the article and approved the submitted version.

## Conflict of Interest

The authors declare that the research was conducted in the absence of any commercial or financial relationships that could be construed as a potential conflict of interest.
